# Gastrointestinal Autonomic Nerve Tumor of the Colon: A Rare Cause of Persistent Abdominal Pain in a Child

**Published:** 2016-01-01

**Authors:** Riccardo Guanà, Elisabetta Teruzzi, Salvatore Garofalo, Isabella Morra, Riccardo Lemini, Jürgen Schleef

**Affiliations:** 1 Department of Pediatric Surgery, Regina Margherita Children's Hospital – Italy; 2 Department of Pathology, Regina Margherita Children's Hospital – Italy

**Keywords:** Gastrointestinal autonomic nerve tumor, Gastrointestinal stromal tumor, Child, Pain abdomen

## Abstract

Gastrointestinal autonomic nerve tumor (GANT) is extremely rare and considered a variant of gastrointestinal stromal tumors (GISTs). GANT originates from the intestinal autonomic nervous system mostly of small intestine or the stomach. We report a colonic GANT diagnosed in a 5-year-old child who presented with abdominal pain and fever for a long period. Colonic resection and end to end anastomosis proved curative without the need of chemo-radiotherapy. Given the rarity of the tumor, the patient is on our long term follow-up.

## INTRODUCTION

GANT is a variant GISTs with ultrastructural features of neural differentiation, and it is also known as plexosarcoma.[1] Clinically it presents with vague abdominal pain, a palpable abdominal mass, nausea or vomiting, hematochezia, anemia, and fever. Immunohistochemistry is required for the diagnosis demonstrating neuron like cells with axonal cytoplasmic processes and neurosecretory granules. Most of the GANTs arise from the small intestine or stomach. Here we report the management of a colonic GANT diagnosed in a young child.

## CASE REPORT

A 5-year-old girl, suffering from persistent abdominal pain and fever for a month, was transferred from another hospital for further management. She complained of severe recurrent diffuse abdominal pain associated with fever. A firm mass was palpable in the left iliac region. Laboratory tests showed neutrophilic leucocytosis and moderate elevation of C-reactive protein, compatible with an infective etiology. Ultrasound abdomen did not provide much information about the nature of the pathology therefore CT scan with contrast was performed which showed a hyperdense mass occupying the lumen of the descending colon. At laparotomy, a 6 cm long intraluminal firm mass was found in the descending colon. The colonic serosa was intact. The colon with the mass was adherent with the peritoneum, omentum, and the kidney capsule. After adhesionolysis, the segmental resection of the colonic portion containing the mass was performed with 2 cm tumor free margins on either side. An end-to-end colo-colonic anastomosis was performed and the resected colon and a small portion of the perirenal fat were sent for histopathological examination. The postoperative course was uneventful and the child was discharged on postoperative day five. The 7-month follow-up was also uneventful.

Histopathological examination showed a diffuse tumour within the muscularis propria, made of either pleomorphic cells with eosinophilic cytoplasm and irregular nuclei or fusiform cells with wavy nuclei. Mitotic activity was very low and surgical margins were negative for tumor cells. At immunohistochemistry, the lesion was positive for Vimentin, S 100, CD 34, Caldesmone; weakly positive for CD 30 and Actin; negative for EMA, CD 117 (c-kit), CD 68, HMB 45, ALK, Cytokeratin, AE1 / AE3, Desmin, CD 21, Neu N (Fig. 1,2). These findings were consistent with a mesenchymal neoplasm originating from the nerve sheaths (so called GANT).

**Figure:**
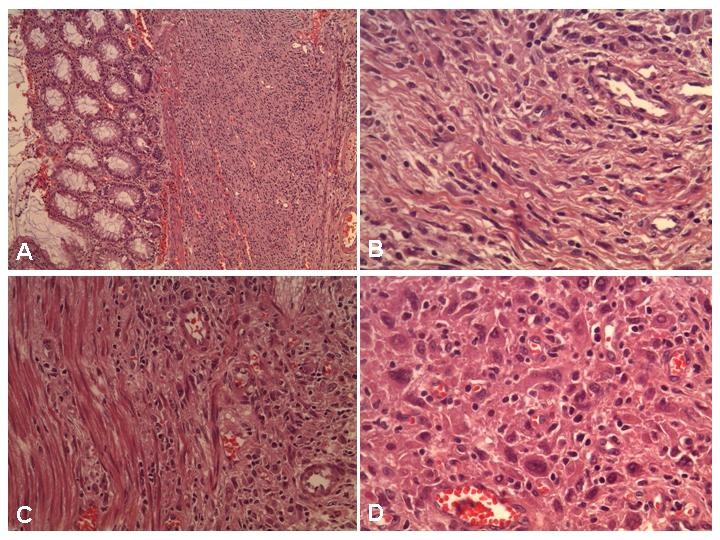
Figure 1:H and E stain: at low magnification sub-mucosal proliferation within the muscularis propria (A-B); at higher magnification epithelioid pleomorphic or fusiform cells (C-D).

**Figure:**
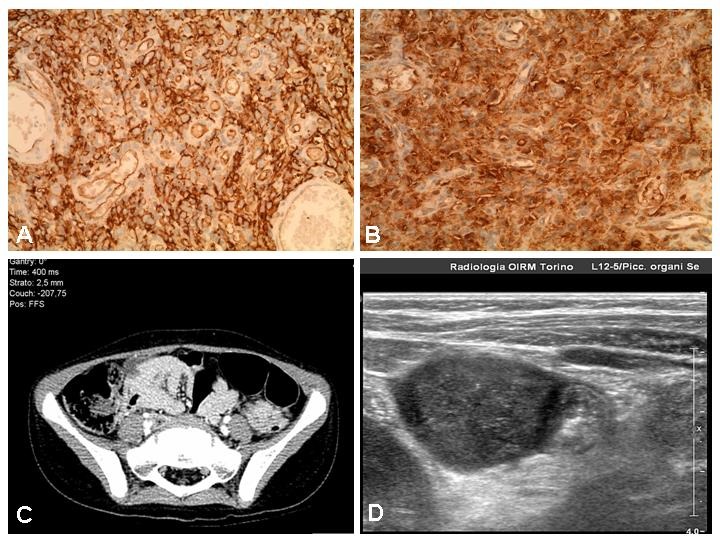
Figure 2:Immunohistochemical positivity for CD 34 (A) and S 100 (B); CT-scan (C) and ultrasound (D) image of the mass.

## DISCUSSION

GANTs usually arise in the small intestine or stomach but other reported sites are in the mesentery and the retroperitoneum.[5,6] These tumors are malignant in the adults especially occurring in stomach with the size more than 5 cm. Six pediatric cases have been described (five females and a male, mean age of presentation 12.5 year) with the primary tumor sited in the stomach in all cases except for the mesenteric involvement in a girl.[1,2] Clinical features at presentation are non-specific: the most common presentation is abdominal pain and fullness, fever, gastrointestinal bleeding, chronic iron deficiency anaemia, weight loss, and malaise. In our case, high grade persistent fever and recurrent diffuse abdominal pain were the dominant presenting features.

At histology the lesion may be spindled or epithelioid and is characterized by complex long thin interdigitating cell processes that are joined together by rudimentary cell junctions. Immunohistochemical features are essentially the same as GIST. Some reports have described chromogranin and synaptophysin staining; Neuron Specific Enolase (NSE) is always positive but is not considered a specific marker.[2,3] The presence of CD34 is reported in all cases, while the S100 protein is present in half of the patients.[2] Muscle markers and vimentin are occasionally reported and genetic studies revealed mutations in KIT gene.[4]

Radical surgical resection with tumor free margins seems curative approach, and long-term survival without recurrence is possible even with large sized tumors.[2] In all pediatric cases, the initial treatment was surgical resection; chemotherapy was administered to one patient on recurrence.[2] However various chemotherapy regimens have been utilized as first-line therapy in adult patients with metastatic/recurrent disease.[5] Radiation therapy has also been utilized in patients with recurrent and/or metastatic disease.[2]

GANTs are extremely rare tumours; in children these are more common in females having smaller lesions and good prognosis with surgical excision compared with adult counterparts. Tumor excision is considered the optimal therapy in children, but a long term follow-up is required to pick the recurrence.

## Footnotes

**Source of Support:** Nil

**Conflict of Interest:** None declared

